# Bronchoalveolar Lavage Fluid-Derived Exosomes: A Novel Role Contributing to Lung Cancer Growth

**DOI:** 10.3389/fonc.2019.00197

**Published:** 2019-04-02

**Authors:** Yibao Yang, Ping Ji, Xuan Wang, Hao Zhou, Junlu Wu, Wenqing Quan, Anquan Shang, Junjun Sun, Chenzheng Gu, Jenni Firrman, Weidong Xiao, Zujun Sun, Dong Li

**Affiliations:** ^1^Department of Clinical Laboratory, Shanghai Tongji Hospital, Tongji University School of Medicine, Shanghai, China; ^2^Department of Pharmacy, Putuo People's Hospital, Shanghai, China; ^3^Dairy and Functional Foods Research Unit, Agriculture Research Service, Eastern Regional Research Center, United States Department of Agriculture, Wyndmoor, PA, United States; ^4^Sol Sherry Thrombosis Research Center, Temple University, Philadelphia, PA, United States

**Keywords:** exosomes, lung cancer, bronchoalveolar lavage fluid, non-typeable *Haemophilus influenza*, tumor necrosis factor alpha

## Abstract

Exosomes are nanovesicles produced by a number of different cell types and regarded as important mediators of cell-to-cell communication. Although bronchoalveolar lavage fluid (BALF) has been shown to be involved in the development of tumors, its role in lung cancer (LC) remains unclear. In this article, we systemically studied BALF-derived exosomes in LC. C57BL/6 mice were injected with Lewis lung carcinoma cells and exposed to non-typeable *Haemophilus influenza* (NTHi) lysate. The analysis showed that the growth of lung tumors in these mice was significantly enhanced compared with the control cohort (only exposure to air). Characterization of the exosomes derived from mouse BALF demonstrated elevated levels of tumor necrosis factor alpha and interleukin-6 in mice exposed to NTHi lysates. Furthermore, abnormal BALF-derived exosomes facilitated the development of LC *in vitro* and *in vivo*. The internalization of the BALF-derived exosomes contributed to the development of LC tumors. Collectively, our data demonstrated that exosomes in BALF are a key factor involved in the growth and progression of lung cancer.

## Introduction

Lung cancer (LC) is a serious public health problem and a major cause of cancer-related death worldwide. Owing to the difficulties in the early detection of LC, the mortality rate among patients remains high, with a 5-year survival rate of merely 18% after diagnosis (1). Pulmonary adenocarcinoma is the major histological type of lung tumors (2). Emerging evidence indicates that the tumor microenvironment (TME) is closely associated with tumor progression (3). Studies investigating the communication between the TME and tumor cells have mostly been limited to soluble factors produced by inflammatory cells in the TME, which may contribute to the development of cancer (4, 5). A growing body of evidence suggests that exosomes mediate cell-to-cell communication (6, 7), and play an important role in tumor progression and metastasis (8–10).

Exosomes are nanoscale vesicles with a size ranging from 30 nm to 150 nm (11–13). In 1983, Johnstone identified these vesicles termed exosomes in culture supernatant and regarded them as a waste product released by erythrocytes (14). Exosomes are secreted by various living cells, including macrophages (15), dendritic cells (16), B cells (17), platelets (18), and tumor cells (19). In addition, exosomes have been found in body fluids, such as saliva (20), urine (21), breast milk (22), plasma (23), and the bronchoalveolar lavage fluid (BALF) (24). Increasing evidence indicates that exosomes play a key role in disease development *in vivo*. Recently, research suggested that BALF-derived exosomes may contribute to inflammation by increasing the level of cytokines (such as interleukin-13, interferon-γ) and the production of leukotriene C4 (24, 25). In addition, Edward et al. demonstrated that exosomes found in breast milk at different periods of time can promote the epithelial-mesenchymal transition of breast cancer cells (26). However, the role of BALF-derived exosomes in LC remains poorly understood.

In the present study, we investigated the contribution of BALF-derived exosomes to the progression of LC using *in vitro* and *in vivo* models.

## Materials and Methods

### Cell Culture and Reagents

Mouse Lewis lung carcinoma (LLC) cells were purchased from the American Type Culture Collection and cultured in Dulbeccos's modified Eagle's medium (DMEM) (Hyclone Laboratories, Inc., South, UT, USA) supplemented with 10% fetal bovine serum (FBS) (Invitrogen, Grand Island, NY, USA), 100 U/mL penicillin and 100 U/mL streptomycin (Hyclone Laboratories, Inc., South, UT, USA), and maintained in a humidified 5% CO_2_ atmosphere at 37°C.

### Construction of Mouse Lung Cancer Model

C57BL/6 male mice (aged 6–8 weeks) were purchased from Shanghai Experimental Animal Slack limited Ltd, qualified SCXK (Shanghai, China) 2012-0002, and maintained at the pathogen-free Central Animal Facility of the Tongji Hospital of Tongji University. Animal experiments were strictly performed according to the Guidelines for the animal care and use issued by the Animals of the National Institutes of Health in Bethesda, MD, USA. All experiments involving animals were approved by the Ethics Committee of Tongji Hospital of Tongji University. A mouse model of LC was generated as previously described (27) with some modifications. In brief, 2 × 10^5^ LLC cells per mouse were injected through the tail vein. After 1 week, the mice were exposed to non-typeable *Haemophilus influenza* (NTHi) lysates or filtered air. The mice were exposed to NTHi lysates for 20 min per time, once per week, for 3 weeks. After anesthesia, the animals were sacrificed, and the lungs were removed and fixed for further experiments.

### Measurement of Inflammatory Cells

Bronchoalveloar lavage fluids (BALF) were collected from mice as previously described (28, 29). The total number of leukocytes in the BALF of mice was counted using a hemocytometer, 50 μL of BALF were centrifugated at 12000 rpm, 4 min by cytospin (Thermo-Fischer, Waltham, MA, USA) to determine the percentage of leukocyte populations through Wright-Giemsa staining.

### Exosome Isolation and Labeling

After tracheal intubation of the mice, the lungs were gently lavaged thrice with 1 mL of ice cold, sterile phosphate-buffered saline (PBS). The fluids collected from mice were maintained at −80°C and immediately processed for exosome isolation.

Exosome isolation was performed through differential ultracentrifugation as previously described (28, 29). The concentration of protein in exosome pellets was measured using the bicinchoninic acid assay (Beyotime, Shanghai, China). For the *in vitro* uptake experiment, exosomes were labeled using a PKH67 green fluorescent cell linker mini kit (Sigma Aldrich, St. Louis, MO, USA) according to the instructions provided by the manufacturer. Ten random fields were counted. For the *in vivo* uptake study, the labeling procedure was identical to that used in the *in vitro* analysis. The labeled exosomes were delivered into the mice, and 18 h later the lungs were removed and embedded in OCT compound, and stored at −80°C. Photographs were captured using an immunofluorescence microscope (Nikon, Tokyo, Japan).

## Preparation of NTHI Lysates

The NTHi lysate was produced from *Haemophilus haemolyticus* as previously described (30). First, the NTHi was inoculated on chocolate agar for 24 h in 5% CO_2_ at 37°C. Then they were grown in 1 L of brain-heart infusion broth (Sigma-Aldrich, St. Louis, MO) containing 3.5 μg/mL NAD (Sigma-Aldrich, St. Louis, MO). Next, the medium was centrifuged at 2500 × g for 10 min at 4°C, washed and resuspended in 20 mL PBS. The culture was transferred to the 100 mm dish and ultraviolet irradiated. Last, the culture was transferred to a 50 mL tube and sonicated three times for 30 s. The concentration of protein was quantified by a bicinchoninic assay (Beyotime, Shanghai, China) and then adjusted to 2.5 mg/mL in PBS and frozen at −80°C.

### Transmission Electron Microscopy (TEM)

A 10 μL aliquote of the exosome suspension was added onto a 200-mesh carbon-coated copper grid for 1 min. Subsequently, the grid was negatively stained with 2% uranyl acetate for 1 min. The sample was observed using a FEI Tecnai G2 spirit transmission electron microscope (Thermo-Fischer, Waltham, MA, USA).

### Nanosight Particle Tracking Analysis (NTA)

For NTA, the size distribution of the purified exosomes was analyzed using the NanoSight LM10 system (Malvern Instruments Ltd. Malvern, UK). In brief, exosome suspension was diluted in PBS. Subsequently, the software of the NanoSight LM10 system was used to determine the concentration and particle size of exosomes.

### Western Blotting Analysis

Western blotting analysis was carried out as previously described (31). Briefly, exosomes were lysed by radio immunoprecipitation assay buffer (RIPA) (CST, Boston, USA) containing protease and phosphatase inhibitor cocktails (Roche). Equal amounts of exosomes were loaded on a 10% SDS polyacrylamide gel. The antibodies used for western blot are as following: CD63, CD9, HSP70, and TSG101 purchased from Santa Cruz (Santa Cruz Biotechnology, Inc., Texas, USA), Calnexin (Abcam plc, Cambridge, UK), PCNA (CST, Boston, USA) HRP-conjugated goat anti-rabbit (Santa Cruz Biotechnology, Inc., Texas, USA) or rabbit-anti-mouse (Dako, Carpinteria. CA, USA) was used as secondary antibody.

### Cell Proliferation Assay

A cell proliferation assay was performed using the cell count kit-8 (DojinDo, Tokyo, Japan). In brief, 1,000 LLC cells were seeded into a 96-well plate and treated with exosomes (5 μg/mL) for 0, 24, 48, and 72 h. Absorbance at 450 nm was measured using a microplate reader (ThermoFisher Scientific, Waltham, MA, USA).

### Cell Migration and Invasion Assay

Invasion and migration assays were performed using a 24-well transwell insert, with 8 μm pores, with or without pre-coated Matrigel (Corning, USA). The LLC cells were trypsinized and washed thrice with serum-free DMEM. A total of 2 × 10^4^ cells were suspended in 100 μL of serum-free DMEM and added to the upper inserts. Exosomes (10 μg/mL), or PBS (control) were added to wells containing DMEM with 2% FBS. The invasion assay was performed in a similar manner, with minor modification. The LLC cells were co-cultured with exosomes (10 μg/mL) or PBS for 24 h, and subsequently trypsinized and washed for the migration assay. A total of 2 × 10^4^ cells were suspended in 500 μL of serum-free DMEM and added to the upper inserts. Seven hundred fifty microliter DMEM containing 10% FBS (750 μL) was added to the wells. After 24 h, the LLC cells on the upper surface of the chamber were removed using a cotton swab, whereas those at the lower membrane surface were fixed with 4% paraformaldehyde and stained with 0.1% crystal violet. Ten random fields were counted.

### Enzyme-Linked Immunosorbent Assay (ELISA)

The levels of tumor necrosis factor-α (TNF-α) and interleukin-6 (IL-6) in the exosomes obtained from mouse BALF were measured using ELISA kits (USCN Life Science & Technology, Missouri City, TX, USA) according to the instructions provided by the manufacturer. In brief, before the ELSIA assay, we used RIPA lysis buffers to lysis exosomes and then we adjusted the concentration of exosome. The remaining steps follow the instructions.

### Animal Experiments

LLC cells (2 × 10^5^ cells per mouse) were injected into C57BL/6 mice via the tail vain. One week later, purified exosomes (5 μg) or PBS were delivered into the mice twice per week for 2 weeks. All mice were sacrificed 4 weeks after injection. Tumor volume (mm^3^) was calculated as (a^2^ × b)/2, where “a” and “b” represent the width and length, respectively.

### Histology and Immunohistochemistry

Lungs were fixed in 10% formalin and embedded in paraffin. Tumor tissues were cut into 4 μm thick sections and stained using a hematoxylin and eosin (H&E) kit, according to the instructions provided by the manufactures. For the immunohistochemical experiment, tumor tissue was incubated with primary antibodies against proliferating cell nuclear antigen (PCNA) overnight at 4°C. Subsequently, the tumor tissues were washed using PBS, and the analysis was performed using the GTvision IHC kit (Gene Tech, Shanghai, China) according to the instructions provided by the manufacture.

### Statistical Analysis

Values are presented as means ± standard error of the mean (SEM). Student's *t*-test (two-sided) was applied for the analysis of differences between two groups. *P* < 0.05 denoted statistical significance. All data were analyzed using the SPSS 22 statistical software (SPSS Inc, Chicago, IL, USA).

## Results

### NTHi Lysates Promote Lung Tumor Growth

**Figure 1:**
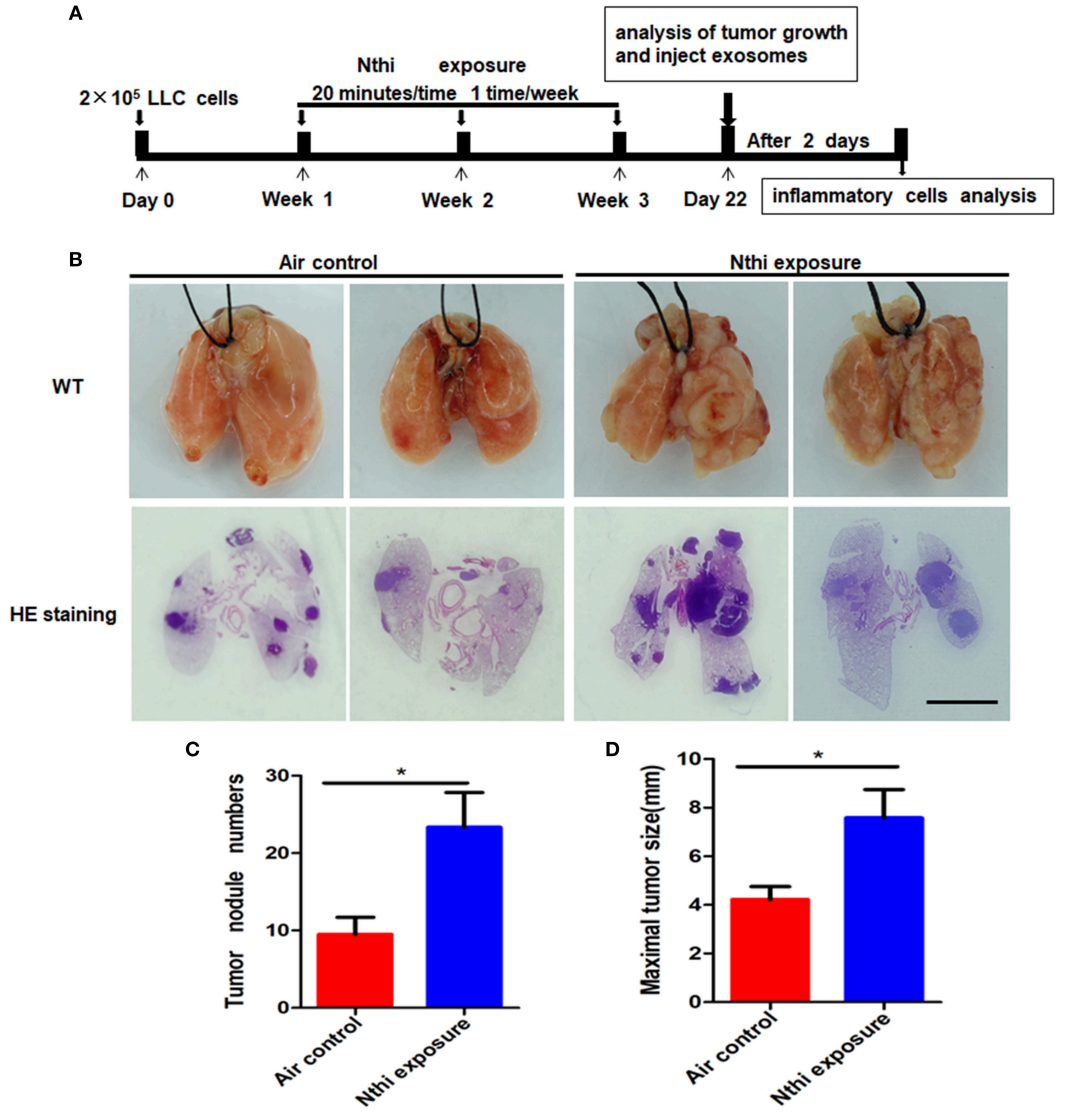
Exposure to NTHi lysate promotes the development of lung tumor. **(A)** Scheme of experimental protocols for the promotion of NTHi-induced lung cancer. **(B)** Lung appearance and histology in WT mice 3 weeks after initiation of the NTHi lysate exposure protocol. Scar bar = 100 μm. **(C,D)** The number of tumor nodules and the maximal tumor diameter detectable on the surface were determined. Results are means ± SEM (*n* = 7), ^*^P < 0.05.

**Figure 2:**
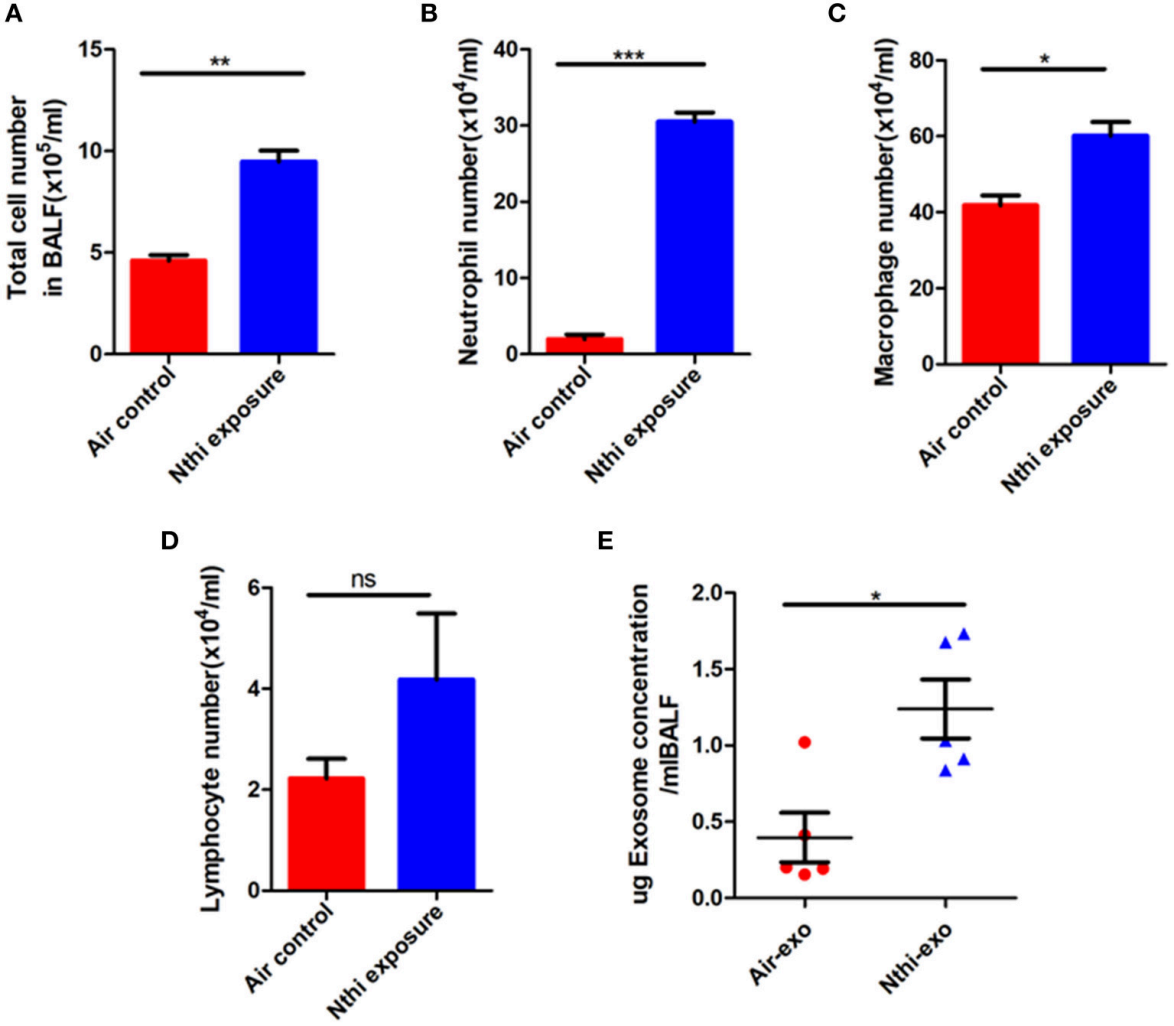
Analysis of the cells and exosomal protein concentration in the bronchoalveolar lavage fluid (BALF). **(A)** Total cell numbers in the BALF of mice exposed to NTHi lysate or air. **(B–D)** The composition of cells was evaluated through cytospin preparations. The number of **(B)** neutrophils, **(C)** macrophages, and **(D)** lymphocytes in the BALF of mice exposed to NTHi lysate or air. **(E)** Concentration of exosomal protein (μg) from BALF of NTHi- and air-induced mice. Results are means ± SEM (*n* = 5), ^*^P < 0.05, ^**^P < 0.01, ^***^P < 0.001.

Inflammation plays a key role in tumor progression (32). We established a metastatic mouse model of LC to better understand the mechanism through which NTHi lysates induce the development of lung neoplasma. Mice were exposed to either 2.5 mg/mL NTHilysates (Figure 1A) or clean air. After 3 weeks, we found that the mice exposed to NTHi lysates exhibited a significant increase in lung tumor growth compared with those exposed to air. H&E staining demonstrated that the tumor nodules were significantly increased, and the diameter of the tumor was larger in mice exposed to NTHi lysates vs. air-exposed mice (Figure 1B). The maximum diameter and number of lung tumor nodules greatly increased in mice treated with NTHi lysates compared with those observed in mice exposed to air (Figures 1C,D). In addition, we found that the number of total leukocyte cells, neutrophils, and macrophages in the BALF increased in mice exposed to NTHi compared with those reported in those exposed to air (Figures 2A–C). However, the number of lymphocytes remained unchanged (Figure 2D). Significant differences were also found in exosomal protein concentration after NTHi exposure vs. air exposure due to the large increase in cell numbers (Figure 2E).

### Characterization of Exosomes From the BALF of Mice

**Figure 3:**
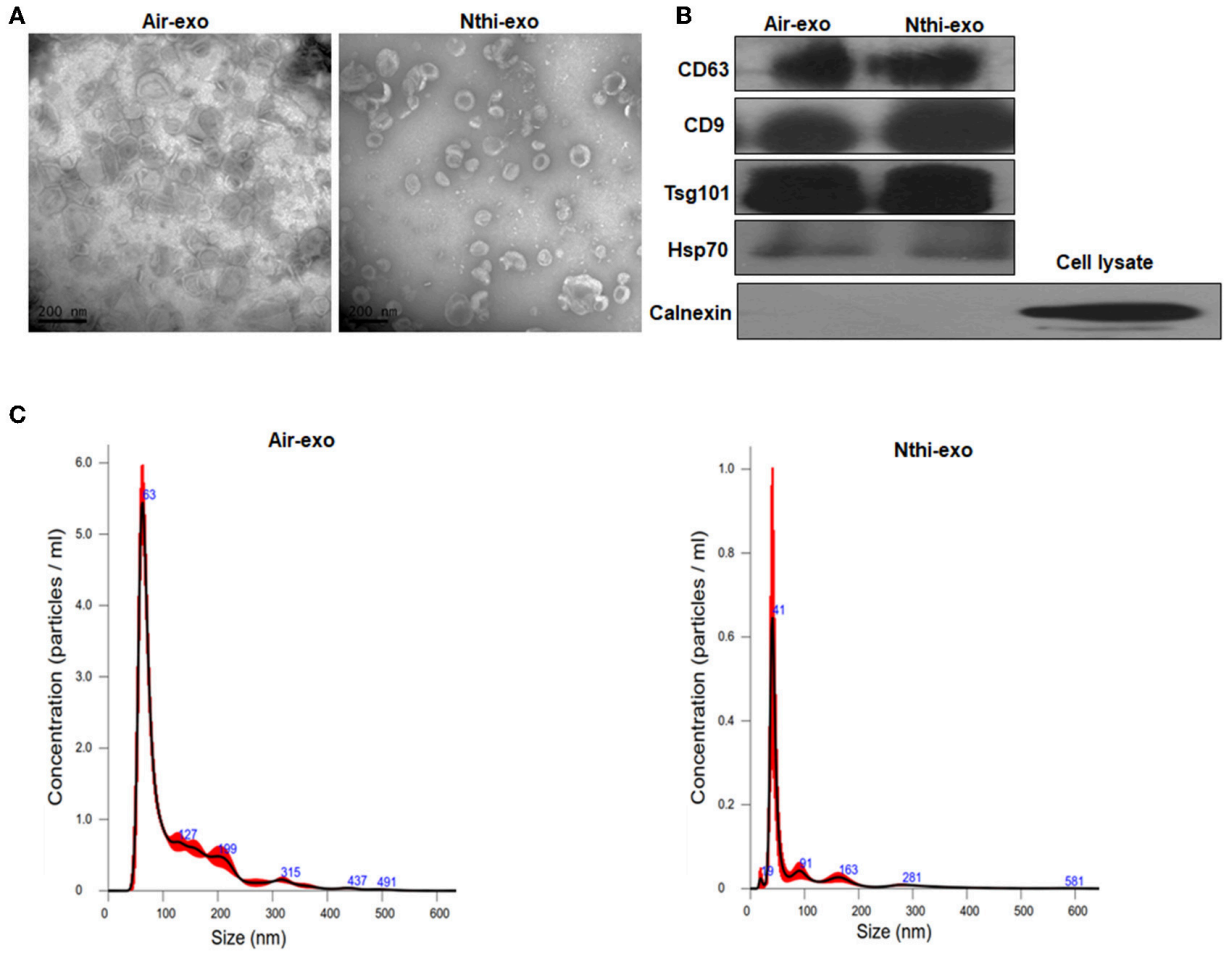
Characterization of exosomes from the BALF of mice exposed to NTHi lysate or air. **(A)** Transmission electron microscopy images of NTHi-Exos and air-Exos. **(B)** Western blotting analysis of exosomal markers CD63, CD9, Hsp70, Tsg101, and negative biomarkers Calnexin. **(C)** Nanoparticle tracking analysis determined the size distribution of NTHi-Exos and air-Exos. Results are presented as means ± SEM of three independent experiments.

Exosomes in the BALF of mice exposed to NTHi lysates or air were purified through differential ultracentrifugation— an established method for the isolation of exosomes (31). The morphology of exosomes was analyzed using TEM. Both BALF-derived air-Exos (i.e., exosomes obtained from mice exposed to air) and NTHi-Exos (i.e., exosomes obtained from mice exposed to NTHi) were typical rounded vesicles ranging from 30 to 150 nm in size (Figure 3A). Western blotting analysis demonstrated that the exosomes were positive for the hallmark exosome proteins, including CD63, CD9, Hsp70, and Tsg101 (28, 29) (Figure 3B). The NTA showed that the peak sizes of air-Exos and NTHi-Exos were 63 nm and 41 nm, respectively (Figure 3C). Collectively, these data indicated that BALF-derived vesicles exhibit the general features associated with exosomes.

### Proinflammatory Cytokines Were Upregulated in NTHi-Exos

**Figure 4:**
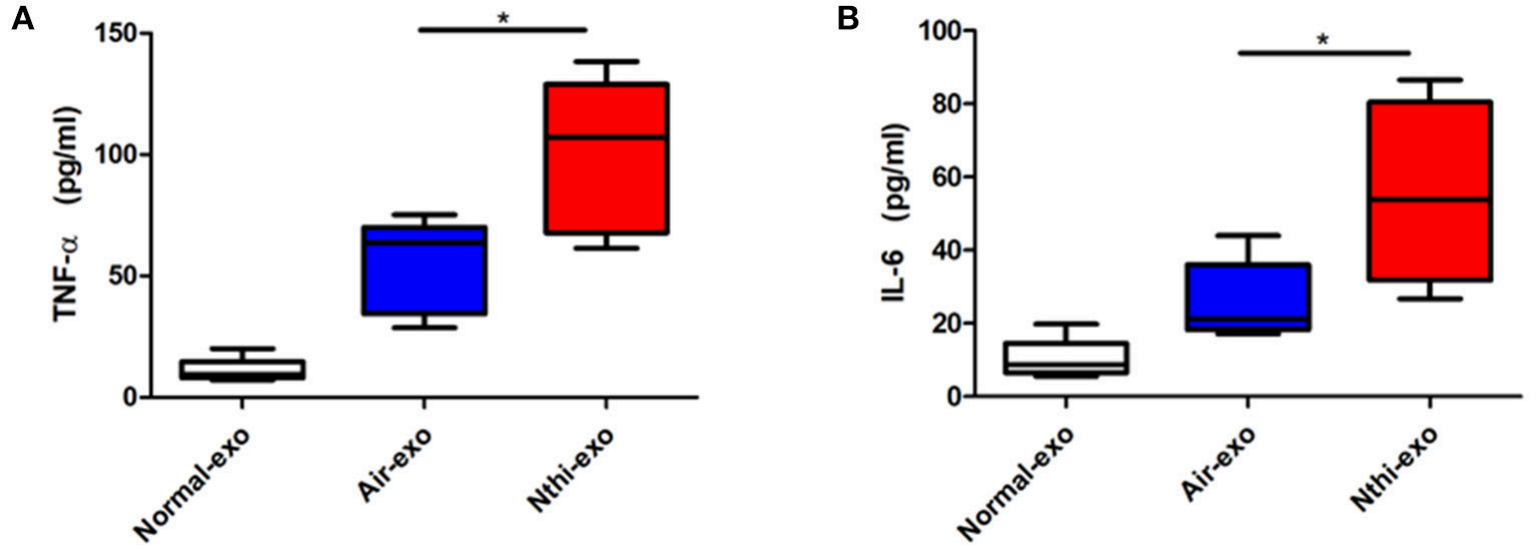
The expression of TNF-αand IL-6in exosomes from the BALF. **(A)** The levels of TNF-α and **(B)** IL-6 were analyzed using ELISA. Results are means ± SEM (*n* = 5, ^*^P < 0.05).

Subsequently, we investigated the cytokine content (i.e., TNF-α and IL-6) of exosomes released in response to exposure to NTHi lysates. The results showed that the levels of TNF-α and IL-6 were upregulated in NTHi-Exos compared vs. air-Exos (Figures 4A,B).

### NTHi-Exos Promote the Proliferation, Migration, and Invasion of LLC Cells

**Figure 5:**
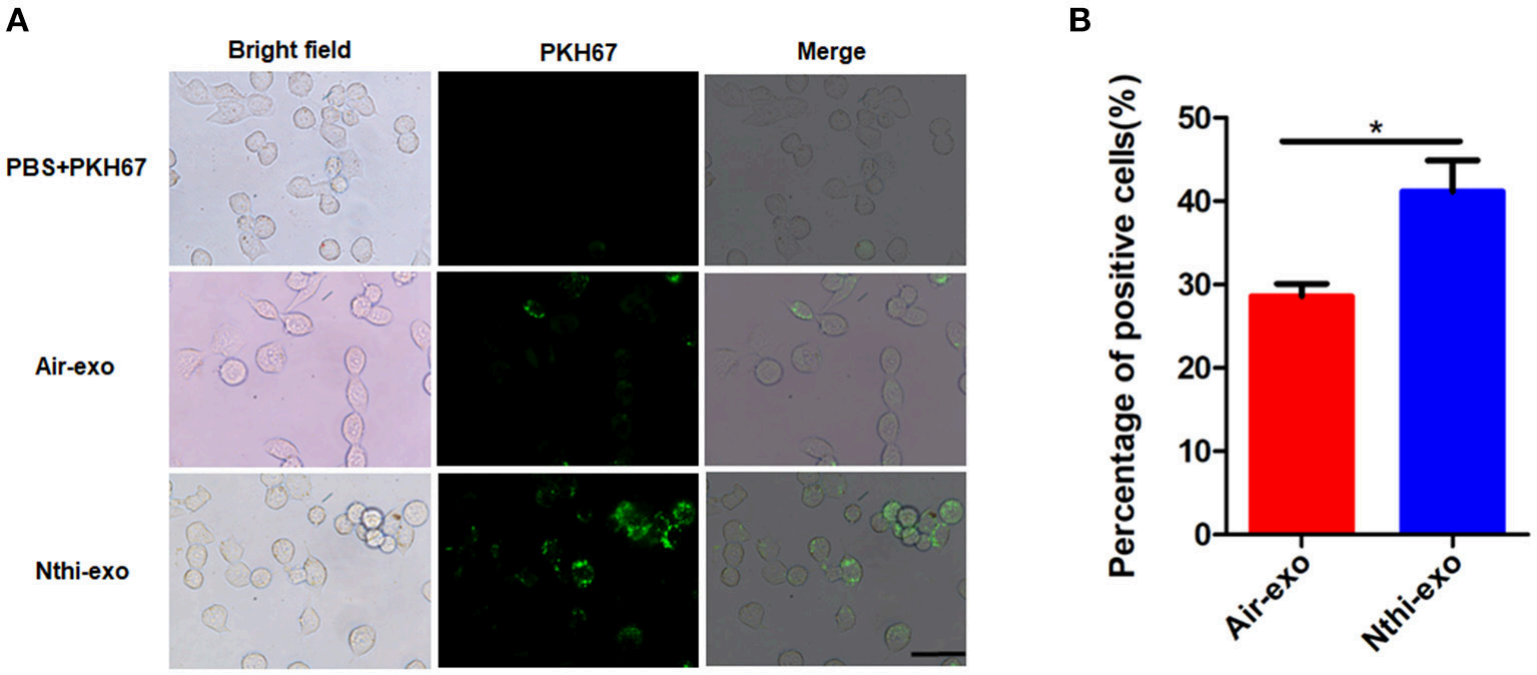
Internalization of exosomes from the BALF of mice exposed to NTHi lysate or air. **(A)** LLC cells demonstrated uptake of labeled-exosomes (green fluorescent dye), images are taken at 24 h co-culture with LC cells. Scar bar = 100 μm. **(B)** the percentage of positive LLC cells engulfing labeled-exosomes (*n* = 10). Results are presented as means ± SEM. (^*^P < 0.05).

**Figure 6:**
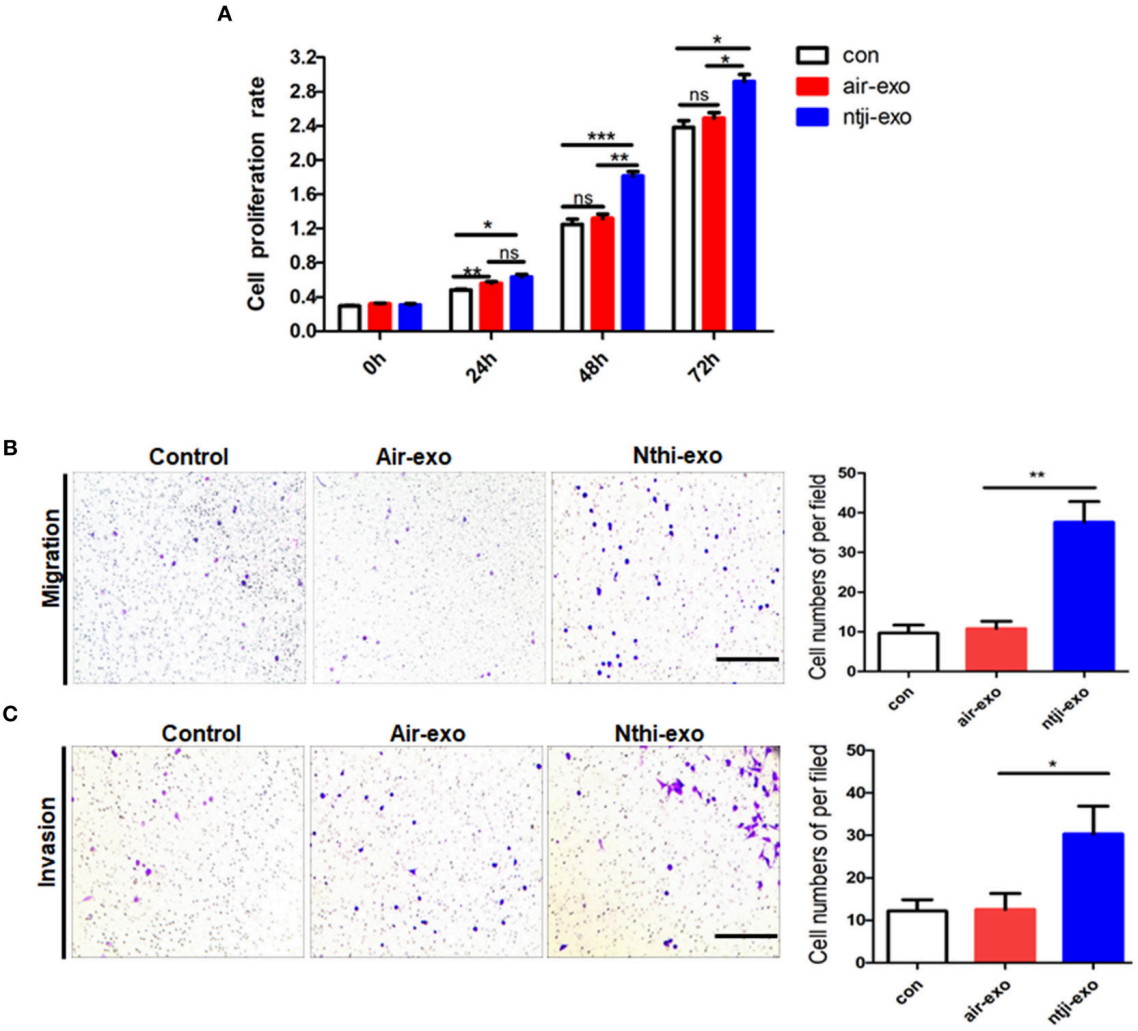
Roles of exosomes from the BALF of mice exposed to NTHi lysate or air. **(A)** Cell proliferation assay was performed in LLC cells incubated with PBS, air-Exosor NTHi-Exos (*n* = 4). **(B)** Migration assay was performed in LLC cells co-cultured with PBS, air-Exos or NTHi-Exos (*n* = 10). **(C)** Invasion assay was performed in LLC cells co-cultured with PBS, air-Exos or NTHi-Exos (*n* = 10). Scar bar = 200 μm. Results are presented as means ± SEM of three independent experiments. (^*^P < 0.05, ^**^P < 0.01, ^***^P < 0.001).

We labeled the exosomes obtained from the BALF of mice using the green fluorescent marker PKH67 and co-cultured them with LLC cells for 24 h to determine the uptake of exosomes by LC cells. The results showed that LLC cells can uptake exosomes (Figure 5A). Meanwhile, we found that the percentage of LLC cells engulfing NTHi-exo is much higher than that of Air-exo (Figure 5B). In order to examine whether BALF-derived NTHi-Exos or air-Exos exerted an effect on LLC cells, we investigated the effect of BALF-derived NTHi-Exos and air-Exos on their proliferation, migration, and invasion. LLC cells co-cultured with NTHi-Exos exhibited higher proliferation rates than those co-cultured with air-Exos or control (Figure 6A). In addition, LLC cells treated with NTHi-Exos showed higher motility than those treated with air-Exos or control (Figure 6B). Similar to the results of migration, cellular invasion was also enhanced in response to the NTHi-Exos condition (Figure 6C). These results revealed that BALF-derived NTHi-Exoscan promote the proliferation, migration, and invasion of LLC cells.

### NTHi-Exos Promote LC Growth *in vivo*

**Figure 7:**
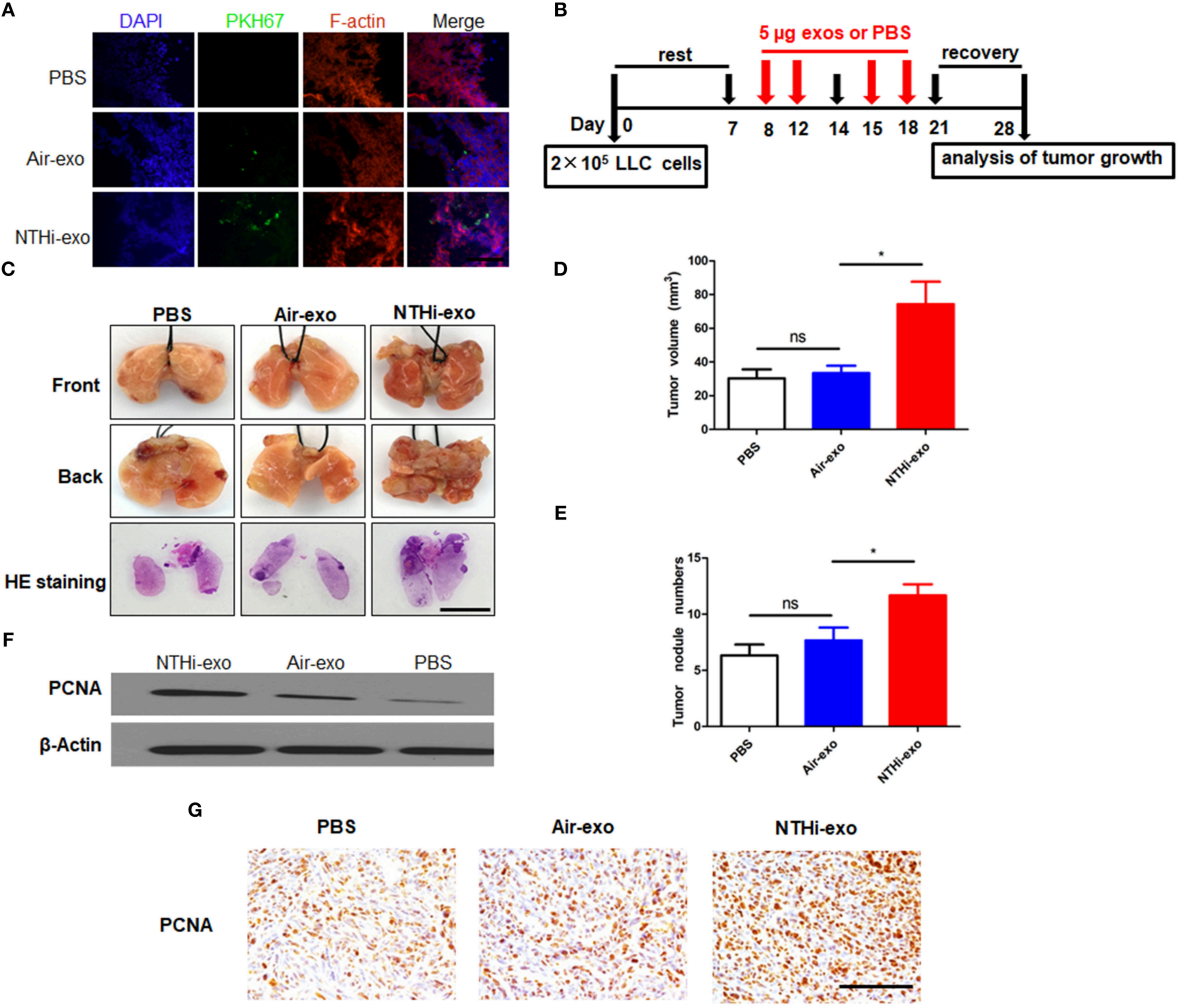
NTHi-Exos promote tumor growth *in vivo*. **(A)** Tumor cells demonstrated uptake ofPKH-67 labeled exosomes from the BALF. Scar bar = 100 μm. **(B)** Scheme of experimental protocols for animal experiment. **(C)** Microscopic and macroscopic analysis of lung cancer after injection of exosomes or PBS control (*n* = 5) Scar bar = 100 μm. **(D–E)** Lung tumor nodules and volume detectable on the surface were determined. **(F)** Western blotting analysis of PCNA protein levels in excised lung tumors treated with NTHi-Exos, air-Exos, and PBS control. **(G)** Immunohistochemical analysis of PCNA protein levels in tumor tissues. Scar bar = 200 μm. Results are presented as means ± SEM of three independent experiments. ^*^P < 0.05.

We used a metastatic mouse model of LC for animal experiments to further examine the role of BALF-derived exosomes on tumor growth *in vivo*. We delivered PKH67-labeled exosomes into C57BL/6 mice using a new intubation method (33). After 18 h, we observed that the exosomes were internalized by tumor cells (Figure 7A). Subsequently, according to the experimental flow chart (Figure 7B), mice were sacrificed 4 weeks later, and the lungs were removed for further analysis. H&E staining showed that the tumor nodules were significantly increased, and the diameter of the tumors was larger in mice treated with NTHi-Exos than in mice treated with air-Exos or control (Figure 7C). The results showed that the number of tumor nodules and volume of the tumors in mice treated with NTHi-Exos were significantly larger vs. those observed in mice treated with air-Exos or control (Figures 7D,E). Moreover, the expression level of PCNA—a marker of proliferation—was highly increased in mice treated with NTHi-Exos (Figures 7F,G). These results suggest that NTHi-Exos can facilitate tumor growth *in vivo*.

## Discussion

The present study identified and characterized BALF-derived exosomes from mice. The data obtained using TEM, NanoSight NTA, and western blotting analysis, clearly demonstrated that BALF-derived exosomes exhibit the typical size, morphology, and markers associated with exosomes. Interestingly, we found that, in mice treated with NTHi lysates, BALF-derived exosomes enhanced the growth, migration, and invasion of LLC cells. Chronic obstructive pulmonary disease (COPD) is an important risk factor for lung cancer, and bacterial pathogens frequently colonize in COPD patients, which always induce chronic inflammation of lung. NTHi—a small Gram-negative bacterium—is most often localized in the airways of patients with chronic obstructive pulmonary disease (30). Therefore, we used NTHi lysates to induce chronic inflammation to investigate how bacterial pathogens contribute to the development of lung cancer. Evidence has shown that inflammation induced by NTHi resulted in lung tumor progression in *KRAS*-dependent mouse models and *Gprc5a*-knockout mouse models (34, 35). In the present study, we stimulated mice with NTHi lysates to simulate the microenvironment of chronic obstructive pulmonary disease. Our analysis yielded a similar phenomenon regarding the progression of lung cancer (Figure 1). The development of tumors in mice stimulated by NTHi-induced inflammation was faster than that observed in mice exposed to air.

Exposure to NTHi lysates led to a significant increase in the number of inflammatory cells and the expression of inflammatory factors in the BALF of mice (36). Moreover, in our study, there was a large increase in the number of inflammatory cells in the BALF of mice following exposure to NTHi, especially neutrophils (Figure 2). Notably, the production of exosomes was highly increased in mice exposed to NTHi lysates. We hypothesize that the increase in exosomes is the result of the observed increase in inflammatory cells infiltrating the lungs.

We showed that exosomes from the BALF of mice can be detected in a mouse model of LC. TEM and NanoSight NTA demonstrated that these exosomes were similar to those reported in previous studies, in terms of morphology and size. Notably, the size of BALF-derived exosomes in this study was slightly smaller than those previously reported (i.e., 41–63 nm vs. 100–150 nm, respectively) (Figure 3C). However, these values were consistent with those reported by other groups (12, 29). Furthermore, consistent with previous studies (29), BALF-derived exosomes contained the hallmark protein components of exosomes, such as tetraspanin CD63, CD9, Hsp70, and Tsg101 (Figure 3B). Collectively, these data clearly demonstrate that exosomes were successfully isolated from the BALF of mice.

Recently, several groups have shown that exosomes play key roles in cancer progression and metastasis (37). To our knowledge, few studies have shown the relationship between BALF-derived exosomes and LC. Different from previous studies in animals and patients, which have focused on the role of single populations or mixed populations of exosomes in BALF in the inflammatory disease of the lung (24, 38), we focused on whether BALF-derived exosomes can have an effect on lung carcinoma. Therefore, we investigated the functional role of BALF-derived exosomes in LLC cells and animals. We found that BALF-derived exosomes are easily internalized by LLC cells (Figure 5A). Our data suggest that the mixed exosomes in the BALF of NTHi-exposed mice can activate LLC cells, resulting in higher cell viability, motility, and invasiveness (Figure 6). Moreover, our *in vivo* analysis yielded similar results. With regard to the molecular level—macroscopic or microscopic—the development of tumor in the mice treated with NTHi-Exos was faster than that observed in mice treated with air-Exos or control (Figures 7C,B,E). The expression level of PCNA was highly increased in the mice treated with NTHi-Exos (Figures 7F,G). We have tested the LLC cells treated directly with NTHi lysate. However, the results showed that there is no effect in migration and invasion of LLC cells stimulated with NTHi lysate (2.5, 5, and 10 μg/μL) (Figure S1). These results suggest that NTHi-Exos can facilitate tumor growth *in vivo*.

Although the mechanism involved in this process remains unknown, exosomes are able to modify the microenvironment by potentiating inflammation (24, 25, 39), which plays a critical role in the development of carcinoma (4). Meanwhile, we found that the levels of TNF-α and IL-6 in exosomes were significantly elevated (Figure 4). These cytokines are important inflammatory factors involved in the progression of tumor formation. Therefore, we speculate that BALF-derived exosomes affect the TME through their proinflammatory cargo, leading to initial tumorigenesis and tumor progression. Of note, exosomes were pelleted at a speed of 100,000 g (31), it is possible that debris from NTHi co-isolated with the exosomes. The tumor progression may be the result of the co-stimulatory effect.

This study clearly demonstrated that purified exosomes from BALF-derived mice exposed to NTHi lysates can facilitate the growth and progression of LC *in vitro* and *in vivo*. Importantly, our findings further stress the role of exosomes as key elements in the course of lung tumor development and as potential new therapeutic targets.

## Author Contributions

DL and ZS designed the study and interpreted the data and amended the manuscript. YY and PJ performed the experiments and drafted the manuscript. XW, HZ, and AS participated in animal testing and flow cytometry analysis. JW, WQ, and CG helped to do the analysis. JF and WX amended the manuscript. All authors read and approved the final version of this manuscript.

### Conflict of Interest Statement

The authors declare that the research was conducted in the absence of any commercial or financial relationships that could be construed as a potential conflict of interest.

## Supplementary Material

Figure S1 LLC treated directly with NTHi lysate. **(A)** Proliferation, **(B)** Migration, **(C)** Invasion.

## Abbreviations

LC: lung cancer
IL-6: interleukin-6
TNF-α: tumor necrosis factor alpha
TME: tumor microenvironment
EMT: epithelial-mesenchymal transition
COPD: chronic obstructive pulmonary disease
BALF: bronchoalveolar lavage fluid
LLC: Lewis lung carcinoma
NTHi: non-typeable *Haemophilus influenza*
air-Exos: exosomes from mice exposed to air
NTHi-Exos: exosomes from mice exposed to NTHi lysates.
