# Supplementary figures and images for: Bronchoalveolar Lavage Fluid-Derived Exosomes: A Novel Role Contributing to Lung Cancer Growth
Source: Front Oncol. 2019 Apr 2;9:197. doi: 10.3389/fonc.2019.00197 (PMC6454045; doi:10.3389/fonc.2019.00197)

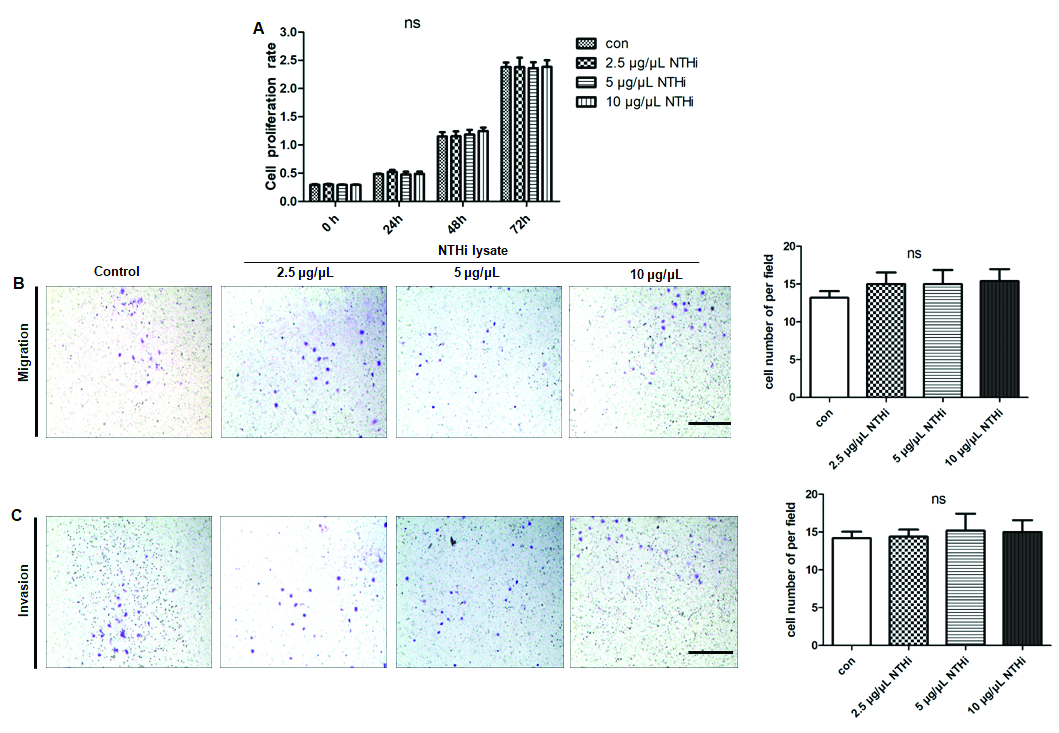

Supplement: Figure S1 — LLC treated directly with NTHi lysate. (A) Proliferation, (B) Migration, (C) Invasion. [file Image_1.tif]
